# 4,8-Dimethyl­pyrano[2,3-*a*]carbazol-2(11*H*)-one

**DOI:** 10.1107/S1600536809009854

**Published:** 2009-03-25

**Authors:** M. Sridharan, K. J. Rajendra Prasad, A. Thomas Gunaseelan, A. Thiruvalluvar, R. J. Butcher

**Affiliations:** a Department of Chemistry, Bharathiar University, Coimbatore 641 046, Tamilnadu, India; b PG Research Department of Physics, Rajah Serfoji Government College (Autonomous), Thanjavur 613 005, Tamilnadu, India; c Department of Chemistry, Howard University, 525 College Street NW, Washington, DC 20059, USA

## Abstract

The mol­ecule of the title compound, C_17_H_13_NO_2_, is nearly planar, the r.m.s. deviation for all non-H atoms excluding the two methyl C atoms being 0.089 Å. Inter­molecular N—H⋯O and C—H⋯O hydrogen bonds are found in the crystal structure. C—H⋯π inter­actions are also found. The H atoms of the methyl group attached to the benzene ring are disordered equally over two positions.

## Related literature

For the synthesis of 2-methyl- and 2-phenyl-pyrano[2,3-*a*]carbazol-4-ones and their derivatives, see: Kavitha & Rajendra Prasad (2003 ▶). For related crystal structures, see: Sridharan *et al.* (2007 ▶); Sridharan *et al.* (2008*a* ▶,*b* ▶); Sridharan *et al.* (2008 ▶).
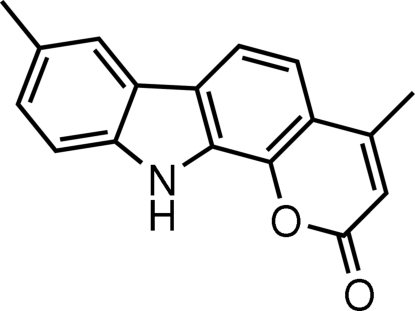


## Experimental

### 

#### Crystal data

C_17_H_13_NO_2_
*M* *_r_* = 263.28
Monoclinic, 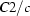

*a* = 26.8502 (4) Å
*b* = 6.8202 (1) Å
*c* = 15.8265 (3) Å
β = 115.531 (2)°
*V* = 2615.21 (9) Å^3^
*Z* = 8
Cu *K*α radiation
μ = 0.71 mm^−1^
*T* = 295 K
0.48 × 0.45 × 0.18 mm

#### Data collection

Oxford Diffraction Gemini R diffractometer
Absorption correction: multi-scan (*CrysAlis RED*; Oxford Diffraction, 2008 ▶) *T*_min_ = 0.313, *T*_max_ = 1.000 (expected range = 0.276–0.880)
6122 measured reflections
2703 independent reflections
2218 reflections with *I* > 2σ(*I*)
*R*_int_ = 0.018

#### Refinement

*R*[*F* ^2^ > 2σ(*F* ^2^)] = 0.044
*wR*(*F* ^2^) = 0.132
*S* = 1.07
2703 reflections
186 parameters
H atoms treated by a mixture of independent and constrained refinement
Δρ_max_ = 0.22 e Å^−3^
Δρ_min_ = −0.18 e Å^−3^

### 

Data collection: *CrysAlis CCD* (Oxford Diffraction, 2008 ▶); cell refinement: *CrysAlis RED* (Oxford Diffraction, 2008 ▶); data reduction: *CrysAlis RED*; program(s) used to solve structure: *SHELXS97* (Sheldrick, 2008 ▶); program(s) used to refine structure: *SHELXL97* (Sheldrick, 2008 ▶); molecular graphics: *ORTEP-3* (Farrugia, 1997 ▶); software used to prepare material for publication: *PLATON* (Spek, 2009 ▶).

## Supplementary Material

Crystal structure: contains datablocks global, I. DOI: 10.1107/S1600536809009854/wn2315sup1.cif

Structure factors: contains datablocks I. DOI: 10.1107/S1600536809009854/wn2315Isup2.hkl

Additional supplementary materials:  crystallographic information; 3D view; checkCIF report

## Figures and Tables

**Table 1:** Hydrogen-bond geometry (Å, °)

*D*—H⋯*A*	*D*—H	H⋯*A*	*D*⋯*A*	*D*—H⋯*A*
N11—H11⋯O2^i^	0.86 (2)	2.01 (2)	2.814 (2)	154.5 (19)
C14—H14*B*⋯O2^ii^	0.96	2.39	3.338 (2)	168
C6—H6⋯*Cg*1^iii^	0.93	2.90	3.389 (1)	114
C5—H5⋯*Cg*2^iii^	0.93	2.98	3.626 (1)	128

Symmetry codes: (i) 
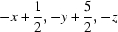
; (ii) 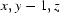; (iii) 
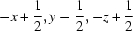
. *Cg*1 is the centroid of the pyrrole ring and *Cg*2 is the centroid of the C6*B*–C10*A* ring.

**Fig. 1.**
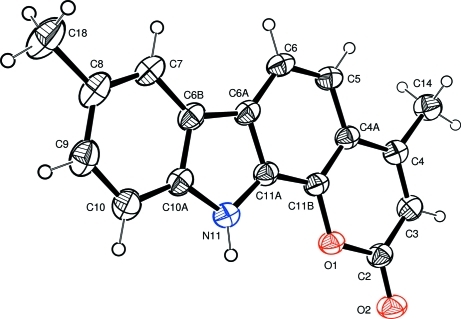
The molecular structure of the title compound, showing the atom-numbering scheme and displacement ellipsoids drawn at the 30% probability level. H atoms are shown as small spheres of arbitrary radius. Only one set of disordered H atoms is shown.

**Fig. 2.**
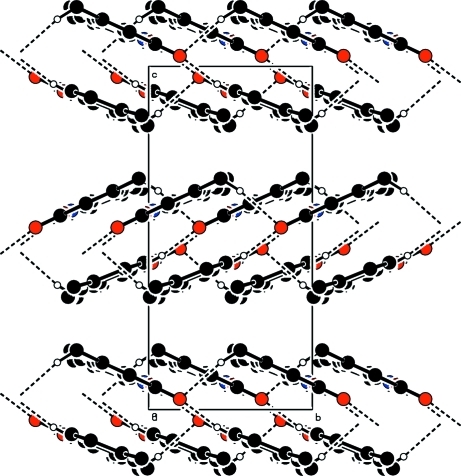
The packing of the title compound, viewed down the a axis. Dashed lines indicate hydrogen bonds. H atoms not involved in hydrogen bonding have been omitted.

## supplementary crystallographic information

### Comment

The title compound has been analysed as part of our crystallographic studies on pyranocarbazolones. Sridharan *et al.* (2007, 2008*a*, 2008*b*), also Sridharan *et al.* (2008) have reported the X-ray crystal sructures of related pyranocarbazolone compounds. Recently we reported (Kavitha & Rajendra Prasad, 2003) the synthesis of 2-methyl- and 2-phenyl- pyrano[2,3-*a*]carbazol-4-ones and their substituted derivatives.

The molecule of the title compound, C_17_H_13_NO_2_, (Fig.1) is nearly planar; the r.m.s. deviation for all non-H atoms excluding the two methyl C atoms is 0.089 Å. N11—H11···O2(1/2 - *x*, 5/2 - *y*, -*z*) and C14—H14B···O2(*x*, -1 + *y*, *z*) intermolecular hydrogen bonds are found in the crystal structure. Furthermore, a C6—H6···π(1/2 - *x*, -1/2 + *y*, 1/2 - *z*) interaction involving the pyrrole ring (C6A,C6B,C10A,N11,C11A) and a C5—H5···π (1/2 - *x*, -1/2 + *y*, 1/2 - *z*) interaction involving the fused benzene ring (C6B–C10A) are also found.

### Experimental

A mixture of 6-methyl-1-hydroxycarbazole (0.199 g, 0.001 mol) and ethyl acetoacetate (0.001 mol) was treated with fused ZnCl_2_/POCl_3_ (1.5 g / 6 ml) and heated to 373 K for 4 h. The reaction was monitored by TLC. After completion of the reaction, the mixture was poured on to crushed ice; the solid that separated was filtered off, dried and recrystallized from ethanol to obtain the title compound as the sole product in high yield (0.267 g, 82%).

### Refinement

H11, attached to N11, was located in a difference Fourier map and refined isotropically; the final N—H distance was 0.86 (2) Å. The remaining H atoms were positioned geometrically and allowed to ride on their parent atoms, with C—H = 0.93 and 0.96 Å for C*sp*^2^ and methyl H atoms, respectively. *U*_iso_(H) = *xU*_eq_(C), where *x* = 1.5 for methyl H atoms and 1.2 for other C-bound H atoms. The H atoms of the C18 methyl group were refined as disordered equally over two positions.

### Crystal data

**Table:**

C_17_H_13_NO_2_	*F*(000) = 1104
*M**_r_* = 263.28	*D*_x_ = 1.337 Mg m^−^^3^
Monoclinic, *C*2/*c*	Melting point: 506(1) K
Hall symbol: -C 2yc	Cu *K*α radiation, λ = 1.54184 Å
*a* = 26.8502 (4) Å	Cell parameters from 3727 reflections
*b* = 6.8202 (1) Å	θ = 5.7–77.3°
*c* = 15.8265 (3) Å	µ = 0.71 mm^−^^1^
β = 115.531 (2)°	*T* = 295 K
*V* = 2615.21 (9) Å^3^	Plate, colourless
*Z* = 8	0.48 × 0.45 × 0.18 mm

### Data collection

**Table:**

Oxford Diffraction Gemini R diffractometer	2703 independent reflections
Radiation source: fine-focus sealed tube	2218 reflections with *I* > 2σ(*I*)
graphite	*R*_int_ = 0.018
Detector resolution: 10.5081 pixels mm^-1^	θ_max_ = 77.8°, θ_min_ = 5.7°
φ and ω scans	*h* = −33→34
Absorption correction: multi-scan (*CrysAlis RED*; Oxford Diffraction, 2008)	*k* = −8→6
*T*_min_ = 0.313, *T*_max_ = 1.000	*l* = −19→18
6122 measured reflections	

### Refinement

**Table:**

Refinement on *F*^2^	Primary atom site location: structure-invariant direct methods
Least-squares matrix: full	Secondary atom site location: difference Fourier map
*R*[*F*^2^ > 2σ(*F*^2^)] = 0.044	Hydrogen site location: inferred from neighbouring sites
*wR*(*F*^2^) = 0.132	H atoms treated by a mixture of independent and constrained refinement
*S* = 1.07	*w* = 1/[σ^2^(*F*_o_^2^) + (0.0834*P*)^2^ + 0.3277*P*] where *P* = (*F*_o_^2^ + 2*F*_c_^2^)/3
2703 reflections	(Δ/σ)_max_ = 0.001
186 parameters	Δρ_max_ = 0.22 e Å^−^^3^
0 restraints	Δρ_min_ = −0.18 e Å^−^^3^

### Special details

**Table:**

Geometry. Bond distances, angles *etc*. have been calculated using the rounded fractional coordinates. All su's are estimated from the variances of the (full) variance-covariance matrix. The cell e.s.d.'s are taken into account in the estimation of distances, angles and torsion angles
Refinement. Refinement of *F*^2^ against ALL reflections. The weighted *R*-factor *wR* and goodness of fit *S* are based on *F*^2^, conventional *R*-factors *R* are based on *F*, with *F* set to zero for negative *F*^2^. The threshold expression of *F*^2^ > 2σ(*F*^2^) is used only for calculating *R*-factors(gt) *etc*. and is not relevant to the choice of reflections for refinement. *R*-factors based on *F*^2^ are statistically about twice as large as those based on *F*, and *R*- factors based on ALL data will be even larger.

### Fractional atomic coordinates and isotropic or equivalent isotropic displacement parameters (Å^2^)

**Table:**

	*x*	*y*	*z*	*U*_iso_*/*U*_eq_	Occ. (<1)
O1	0.29530 (4)	1.01921 (13)	0.07257 (7)	0.0519 (3)	
O2	0.35050 (5)	1.19047 (16)	0.03063 (9)	0.0686 (4)	
N11	0.19050 (5)	0.97178 (18)	0.07986 (8)	0.0519 (3)	
C2	0.34394 (6)	1.0397 (2)	0.06525 (10)	0.0537 (4)	
C3	0.38333 (6)	0.8836 (2)	0.10052 (10)	0.0556 (4)	
C4	0.37356 (5)	0.7174 (2)	0.13688 (9)	0.0514 (4)	
C4A	0.32087 (5)	0.69352 (19)	0.13969 (9)	0.0481 (4)	
C5	0.30472 (6)	0.5221 (2)	0.17213 (9)	0.0543 (4)	
C6	0.25386 (6)	0.5074 (2)	0.17191 (9)	0.0545 (4)	
C6A	0.21696 (5)	0.6649 (2)	0.13986 (9)	0.0487 (4)	
C6B	0.16178 (6)	0.7006 (2)	0.12955 (9)	0.0516 (4)	
C7	0.12374 (6)	0.5877 (3)	0.14701 (10)	0.0605 (5)	
C8	0.07214 (6)	0.6638 (3)	0.12677 (11)	0.0691 (5)	
C9	0.05908 (6)	0.8535 (3)	0.09007 (11)	0.0696 (6)	
C10	0.09551 (6)	0.9699 (3)	0.07195 (11)	0.0620 (5)	
C10A	0.14709 (5)	0.8902 (2)	0.09203 (9)	0.0517 (4)	
C11A	0.23240 (5)	0.83643 (18)	0.10748 (9)	0.0469 (4)	
C11B	0.28383 (5)	0.84912 (18)	0.10697 (8)	0.0462 (4)	
C14	0.41674 (6)	0.5602 (3)	0.17296 (12)	0.0658 (5)	
C18	0.02959 (8)	0.5430 (4)	0.14272 (16)	0.0991 (9)	
H3	0.41717	0.89775	0.09821	0.0667*	
H5	0.32922	0.41756	0.19401	0.0651*	
H6	0.24386	0.39332	0.19294	0.0653*	
H7	0.13310	0.46234	0.17205	0.0726*	
H9	0.02434	0.90351	0.07730	0.0835*	
H10	0.08598	1.09560	0.04748	0.0744*	
H11	0.1888 (7)	1.081 (3)	0.0516 (13)	0.070 (5)*	
H14A	0.44916	0.60240	0.16691	0.0988*	
H14B	0.40281	0.44234	0.13739	0.0988*	
H14C	0.42590	0.53528	0.23769	0.0988*	
H18A	−0.00362	0.61818	0.12505	0.1487*	0.500
H18B	0.04377	0.50829	0.20772	0.1487*	0.500
H18C	0.02168	0.42592	0.10550	0.1487*	0.500
H18D	0.04484	0.41674	0.16713	0.1487*	0.500
H18E	−0.00255	0.52663	0.08446	0.1487*	0.500
H18F	0.01954	0.60901	0.18668	0.1487*	0.500

### Atomic displacement parameters (Å^2^)

**Table:**

	*U*^11^	*U*^22^	*U*^33^	*U*^12^	*U*^13^	*U*^23^
O1	0.0547 (5)	0.0430 (5)	0.0602 (5)	−0.0053 (4)	0.0269 (4)	0.0030 (4)
O2	0.0681 (6)	0.0510 (6)	0.0928 (8)	−0.0093 (4)	0.0404 (6)	0.0104 (5)
N11	0.0551 (6)	0.0481 (6)	0.0560 (6)	−0.0014 (5)	0.0272 (5)	0.0044 (5)
C2	0.0561 (7)	0.0484 (7)	0.0580 (8)	−0.0117 (6)	0.0259 (6)	−0.0030 (6)
C3	0.0475 (6)	0.0582 (8)	0.0595 (8)	−0.0086 (6)	0.0216 (6)	−0.0022 (6)
C4	0.0502 (7)	0.0539 (7)	0.0453 (6)	−0.0042 (5)	0.0160 (5)	−0.0018 (5)
C4A	0.0526 (7)	0.0481 (7)	0.0410 (6)	−0.0042 (5)	0.0178 (5)	0.0003 (5)
C5	0.0599 (8)	0.0501 (7)	0.0503 (7)	0.0015 (6)	0.0214 (6)	0.0090 (5)
C6	0.0647 (8)	0.0506 (7)	0.0483 (7)	−0.0050 (6)	0.0246 (6)	0.0095 (5)
C6A	0.0554 (7)	0.0514 (7)	0.0405 (6)	−0.0081 (5)	0.0218 (5)	0.0006 (5)
C6B	0.0551 (7)	0.0593 (8)	0.0413 (6)	−0.0089 (6)	0.0216 (5)	−0.0007 (5)
C7	0.0614 (8)	0.0707 (9)	0.0519 (7)	−0.0122 (7)	0.0268 (6)	0.0055 (7)
C8	0.0588 (8)	0.0958 (12)	0.0556 (8)	−0.0151 (8)	0.0273 (7)	0.0064 (8)
C9	0.0526 (7)	0.0983 (13)	0.0605 (9)	0.0001 (8)	0.0268 (7)	0.0068 (8)
C10	0.0580 (8)	0.0713 (9)	0.0593 (8)	0.0015 (7)	0.0277 (6)	0.0046 (7)
C10A	0.0545 (7)	0.0574 (7)	0.0459 (6)	−0.0063 (6)	0.0241 (5)	−0.0014 (5)
C11A	0.0536 (7)	0.0455 (6)	0.0419 (6)	−0.0041 (5)	0.0208 (5)	−0.0009 (5)
C11B	0.0537 (7)	0.0436 (6)	0.0413 (6)	−0.0071 (5)	0.0205 (5)	−0.0009 (4)
C14	0.0527 (7)	0.0678 (9)	0.0709 (9)	0.0052 (7)	0.0210 (7)	0.0094 (7)
C18	0.0691 (10)	0.137 (2)	0.0963 (14)	−0.0196 (12)	0.0406 (10)	0.0270 (14)

### Geometric parameters (Å, °)

**Table:**

O1—C2	1.367 (2)	C8—C18	1.515 (3)
O1—C11B	1.3717 (16)	C9—C10	1.382 (3)
O2—C2	1.2132 (18)	C10—C10A	1.391 (2)
N11—C10A	1.378 (2)	C11A—C11B	1.387 (2)
N11—C11A	1.3730 (19)	C3—H3	0.9300
N11—H11	0.86 (2)	C5—H5	0.9300
C2—C3	1.433 (2)	C6—H6	0.9300
C3—C4	1.347 (2)	C7—H7	0.9300
C4—C4A	1.444 (2)	C9—H9	0.9300
C4—C14	1.500 (2)	C10—H10	0.9300
C4A—C11B	1.3927 (19)	C14—H14A	0.9600
C4A—C5	1.418 (2)	C14—H14B	0.9600
C5—C6	1.368 (2)	C14—H14C	0.9600
C6—C6A	1.400 (2)	C18—H18A	0.9600
C6A—C11A	1.4093 (19)	C18—H18B	0.9600
C6A—C6B	1.440 (2)	C18—H18C	0.9600
C6B—C7	1.399 (2)	C18—H18D	0.9600
C6B—C10A	1.4066 (19)	C18—H18E	0.9600
C7—C8	1.382 (3)	C18—H18F	0.9600
C8—C9	1.400 (3)		
			
O1···N11	2.8837 (19)	C5···H14C	2.9600
O2···N11^i^	2.8136 (17)	C5···H14B	2.9600
O2···C14^ii^	3.338 (2)	C6A···H6^v^	2.8500
O1···H11	2.77 (2)	C6B···H5^v^	3.0800
O2···H14B^ii^	2.3900	C9···H14C^v^	2.8600
O2···H10^i^	2.9000	C11A···H6^v^	2.9700
O2···H11^i^	2.01 (2)	C14···H5	2.6900
N11···O1	2.8837 (19)	C18···H3^vii^	2.9600
N11···O2^i^	2.8136 (17)	H3···H14A	2.2700
N11···C4^iii^	3.3621 (17)	H3···C18^viii^	2.9600
C2···C6^iii^	3.548 (2)	H3···H18A^viii^	2.4900
C2···C6A^iii^	3.2532 (19)	H3···H18E^viii^	2.4200
C2···C6B^iii^	3.436 (2)	H5···C14	2.6900
C3···C10A^iii^	3.3608 (19)	H5···H14B	2.5000
C3···C6B^iii^	3.3546 (19)	H5···H14C	2.5100
C4···N11^iii^	3.3621 (17)	H5···C6B^iv^	3.0800
C4···C10A^iii^	3.4985 (18)	H6···C6A^iv^	2.8500
C4A···C11A^iii^	3.5450 (18)	H6···C11A^iv^	2.9700
C5···C10A^iv^	3.5003 (18)	H7···H18D	2.3600
C6···C6A^iv^	3.5963 (19)	H9···H18A	2.3300
C6···C11A^iv^	3.5463 (19)	H9···H9^ix^	2.5800
C6···C2^iii^	3.548 (2)	H10···O2^i^	2.9000
C6A···C6^v^	3.5963 (19)	H11···O1	2.77 (2)
C6A···C2^iii^	3.2532 (19)	H11···O2^i^	2.01 (2)
C6B···C2^iii^	3.436 (2)	H11···C2^i^	3.08 (2)
C6B···C3^iii^	3.3546 (19)	H14A···H3	2.2700
C10A···C4^iii^	3.4985 (18)	H14B···O2^vi^	2.3900
C10A···C3^iii^	3.3608 (19)	H14B···C5	2.9600
C10A···C5^v^	3.5003 (18)	H14B···H5	2.5000
C11A···C6^v^	3.5463 (19)	H14C···C5	2.9600
C11A···C11B^iii^	3.4692 (18)	H14C···H5	2.5100
C11A···C4A^iii^	3.5450 (18)	H14C···C9^iv^	2.8600
C11B···C11B^iii^	3.3601 (16)	H18A···H9	2.3300
C11B···C11A^iii^	3.4692 (18)	H18A···H3^vii^	2.4900
C14···O2^vi^	3.338 (2)	H18B···C3^iv^	2.9400
C2···H11^i^	3.08 (2)	H18D···H7	2.3600
C3···H18B^v^	2.9400	H18E···H3^vii^	2.4200
			
C2—O1—C11B	120.41 (11)	C4—C3—H3	119.00
C10A—N11—C11A	108.09 (12)	C4A—C5—H5	119.00
C11A—N11—H11	126.9 (14)	C6—C5—H5	119.00
C10A—N11—H11	124.3 (14)	C5—C6—H6	120.00
O1—C2—C3	117.55 (13)	C6A—C6—H6	120.00
O1—C2—O2	117.06 (14)	C6B—C7—H7	120.00
O2—C2—C3	125.38 (17)	C8—C7—H7	120.00
C2—C3—C4	122.99 (16)	C8—C9—H9	119.00
C4A—C4—C14	120.98 (13)	C10—C9—H9	119.00
C3—C4—C14	120.22 (15)	C9—C10—H10	122.00
C3—C4—C4A	118.81 (13)	C10A—C10—H10	122.00
C4—C4A—C5	124.12 (13)	C4—C14—H14A	109.00
C4—C4A—C11B	117.05 (12)	C4—C14—H14B	109.00
C5—C4A—C11B	118.82 (14)	C4—C14—H14C	109.00
C4A—C5—C6	121.36 (13)	H14A—C14—H14B	109.00
C5—C6—C6A	119.64 (13)	H14A—C14—H14C	109.00
C6—C6A—C11A	119.71 (14)	H14B—C14—H14C	109.00
C6B—C6A—C11A	105.55 (12)	C8—C18—H18A	109.00
C6—C6A—C6B	134.74 (13)	C8—C18—H18B	109.00
C7—C6B—C10A	119.59 (15)	C8—C18—H18C	109.00
C6A—C6B—C7	133.48 (14)	C8—C18—H18D	109.00
C6A—C6B—C10A	106.92 (13)	C8—C18—H18E	109.00
C6B—C7—C8	119.48 (17)	C8—C18—H18F	109.00
C9—C8—C18	119.98 (17)	H18A—C18—H18B	109.00
C7—C8—C18	120.65 (19)	H18A—C18—H18C	109.00
C7—C8—C9	119.36 (17)	H18A—C18—H18D	141.00
C8—C9—C10	122.95 (17)	H18A—C18—H18E	56.00
C9—C10—C10A	116.87 (17)	H18A—C18—H18F	56.00
N11—C10A—C10	128.99 (14)	H18B—C18—H18C	109.00
N11—C10A—C6B	109.27 (13)	H18B—C18—H18D	56.00
C6B—C10A—C10	121.74 (15)	H18B—C18—H18E	141.00
N11—C11A—C11B	129.60 (12)	H18B—C18—H18F	56.00
C6A—C11A—C11B	120.23 (12)	H18C—C18—H18D	56.00
N11—C11A—C6A	110.17 (13)	H18C—C18—H18E	56.00
C4A—C11B—C11A	120.23 (12)	H18C—C18—H18F	141.00
O1—C11B—C4A	123.06 (13)	H18D—C18—H18E	109.00
O1—C11B—C11A	116.71 (12)	H18D—C18—H18F	109.00
C2—C3—H3	118.00	H18E—C18—H18F	109.00
			
C11B—O1—C2—O2	176.99 (12)	C6—C6A—C6B—C7	−1.0 (3)
C11B—O1—C2—C3	−4.14 (18)	C6—C6A—C6B—C10A	−179.50 (15)
C2—O1—C11B—C4A	2.84 (18)	C11A—C6A—C6B—C7	178.38 (15)
C2—O1—C11B—C11A	−176.72 (12)	C11A—C6A—C6B—C10A	−0.12 (14)
C11A—N11—C10A—C6B	0.99 (15)	C6—C6A—C11A—N11	−179.77 (12)
C11A—N11—C10A—C10	−178.52 (14)	C6—C6A—C11A—C11B	0.25 (19)
C10A—N11—C11A—C6A	−1.08 (15)	C6B—C6A—C11A—N11	0.74 (15)
C10A—N11—C11A—C11B	178.89 (13)	C6B—C6A—C11A—C11B	−179.24 (12)
O1—C2—C3—C4	2.4 (2)	C6A—C6B—C7—C8	−178.08 (15)
O2—C2—C3—C4	−178.81 (15)	C10A—C6B—C7—C8	0.3 (2)
C2—C3—C4—C4A	0.7 (2)	C6A—C6B—C10A—N11	−0.53 (15)
C2—C3—C4—C14	−179.72 (14)	C6A—C6B—C10A—C10	179.03 (13)
C3—C4—C4A—C5	176.95 (13)	C7—C6B—C10A—N11	−179.27 (12)
C3—C4—C4A—C11B	−2.11 (19)	C7—C6B—C10A—C10	0.3 (2)
C14—C4—C4A—C5	−2.6 (2)	C6B—C7—C8—C9	−0.7 (2)
C14—C4—C4A—C11B	178.34 (13)	C6B—C7—C8—C18	178.49 (16)
C4—C4A—C5—C6	−179.13 (13)	C7—C8—C9—C10	0.6 (3)
C11B—C4A—C5—C6	−0.09 (19)	C18—C8—C9—C10	−178.59 (17)
C4—C4A—C11B—O1	0.40 (18)	C8—C9—C10—C10A	−0.1 (2)
C4—C4A—C11B—C11A	179.94 (13)	C9—C10—C10A—N11	179.08 (14)
C5—C4A—C11B—O1	−178.72 (11)	C9—C10—C10A—C6B	−0.4 (2)
C5—C4A—C11B—C11A	0.83 (18)	N11—C11A—C11B—O1	−1.3 (2)
C4A—C5—C6—C6A	−0.6 (2)	N11—C11A—C11B—C4A	179.12 (13)
C5—C6—C6A—C6B	179.79 (14)	C6A—C11A—C11B—O1	178.66 (11)
C5—C6—C6A—C11A	0.48 (19)	C6A—C11A—C11B—C4A	−0.91 (19)

Symmetry codes: (i) −*x*+1/2, −*y*+5/2, −*z*; (ii) *x*, *y*+1, *z*; (iii) −*x*+1/2, −*y*+3/2, −*z*; (iv) −*x*+1/2, *y*−1/2, −*z*+1/2; (v) −*x*+1/2, *y*+1/2, −*z*+1/2; (vi) *x*, *y*−1, *z*; (vii) *x*−1/2, *y*−1/2, *z*; (viii) *x*+1/2, *y*+1/2, *z*; (ix) −*x*, −*y*+2, −*z*.

### Hydrogen-bond geometry (Å, °)

**Table:**

*D*—H···*A*	*D*—H	H···*A*	*D*···*A*	*D*—H···*A*
N11—H11···O2^i^	0.86 (2)	2.01 (2)	2.814 (2)	154.5 (19)
C14—H14B···O2^vi^	0.96	2.39	3.338 (2)	168
C6—H6···Cg1^iv^	0.93	2.90	3.389 (1)	114
C5—H5···Cg2^iv^	0.93	2.98	3.626 (1)	128

Symmetry codes: (i) −*x*+1/2, −*y*+5/2, −*z*; (vi) *x*, *y*−1, *z*; (iv) −*x*+1/2, *y*−1/2, −*z*+1/2.
